# Role and Function of Regulatory T Cell in Chronic Rhinosinusitis with Nasal Polyposis

**DOI:** 10.1155/2022/1144563

**Published:** 2022-03-26

**Authors:** Chenyang Lei, Juan Jiang, Yanyan Zhang, Gaoyun Xiong

**Affiliations:** Department of Otorhinolaryngology, Tongde Hospital of Zhejiang Province, Hangzhou, China

## Abstract

Chronic rhinosinusitis with nasal polyps (CRSwNP) is a subtype of chronic rhinosinusitis characterized by high edema in the stroma, albumin deposition, and formation of pseudocysts. The pathogenesis of CRSwNP is not yet fully understood. Regulatory T (Treg) cells are a subset of CD4+ T cells that play a suppressive immunoregulatory role in the process of CRSwNP. Recent studies have found that there was a significant reduction in Treg cells in polyp tissues, which leads to the onset of CRSwNP. An imbalance between Th17 and Treg cells can also aggravate inflammation toward the Th2 type. This review focuses on our understanding of the function and role of Treg cells and their regulatory factors and clinical significance in CRSwNP. We also summarize the current drug treatments for CRSwNP with Tregs as the potential therapeutic target, which will provide new ideas for the treatment of CRSwNP in the future.

## 1. Introduction

Chronic rhinosinusitis with nasal polyps (CRSwNP) is a subtype of chronic rhinosinusitis (CRS), which is characterized by persistent inflammation of the paranasal sinus mucosa [1]. It is one of the most common nasal diseases that affects a large proportion of the world's population and is reported to be associated with some lower airway diseases, such as asthma [2]. CRSwNP not only causes nasal discomfort such as nasal congestion and purulent discharge but also causes extranasal symptoms such as headache, ear tightness, and sleep disorder [3]. Aside from physical symptoms, CRSwNP can also cause mental symptoms, such as anxiety and depression, which may seriously affect the efficiency of people's lives and work [4, 5].

CRSwNP is characterized by high edema in the stroma, accompanied by albumin deposition and the formation of pseudocysts [6]. At present, it is believed that the colonization of some pathogenic bacteria (such as Staphylococcus aureus) and fungi (such as Aspergillus fumigatus) induces the development of nasal polyposis in CRSwNP [7–9] which is associated with T helper 2 (Th2) inflammation. Moreover, previous studies have reported that the expression of interleukin (IL)-4, -5, -6, and -13 mediated the inflammatory process [10, 11]. In addition, a low frequency of CD4+ T cells and a high frequency of CD8+ T cells were observed in patients with nasal polyposis [12].

During the recruitment of CD4+ T cells into the nasal mucosa, regulatory T (Treg) cells are considered to play a key role in the formation of nasal polyps by modulating the balance of Th1 and Th2 immunity [13, 14]. Studies have been conducted to investigate the function of Tregs. Some of the findings have shown that Tregs are associated with the tolerance to autoantigens and antigens of food and commensal microflora [15–17]. In addition, the reduction in the number or function of peripheral Tregs leads to chronic immunopathological processes, such as cancers and autoimmune disorders [16, 18–20]. This review was aimed at introducing the classification of Tregs, their mechanisms, and functions in the etiology of CRSwNP and Treg as a recent therapeutic target in CRSwNP treatment.

## 2. Subtypes of Treg

Tregs are identified by surface markers (such as CD4 and CD25), intracellular markers (such as Forkhead-Box-Protein P3 (FOXP3)), and cytokines (such as TGF-*β* and IL-10). FOXP3, which is the most important transcription factor, is associated with Treg and CD25 expressions [21, 22]. In addition, the different FOXP3 expression stabilities are an indication to distinguish different types of Treg [23, 24]. The phenotype of Tregs is commonly CD4^+^CD25^+^FOXP3^+^ T cells. However, studies have found that CD8^+^ T cells have immunosuppressive effects in some diseases and organ transplantations [25]. A recent study showed that CD8^+^CD25^+^T cells with immune regulatory activity can also express FOXP3, which is later referred to as CD8 Treg [26, 27]. A study by Pant et al. [28] showed that the reduction of CD8 Treg is associated with the process of CRSwNP.

For Treg subtypes, Tregs can be divided into two categories according to their origin. Tregs derived from the thymus are called thymus-derived Tregs (tTregs), and Tregs that differentiate in peripheral tissues are called peripherally derived Tregs (pTregs) [29]. tTregs express FOXP3 and have high self-affinity for T cell receptors [24]. They are associated with tolerance to autoantigens [16]. pTregs, which are derived from peripheral naive Th cells, are most commonly found in peripheral barrier tissue and act as suppressors of local inflammation when faced with exogenous antigens [16, 30]. The decrease in pTregs may lead to a series of chronic T cell-related immune diseases [30].

Based on differentiation, Tregs can be divided into naive Tregs (nTregs), central memory Tregs (cmTregs), effector memory Tregs (emTregs), and effector Tregs (eTregs) [24]. Each has different actions in the lymphoid or lymphoid organs. Their differentiation and homing direction require specific receptors and cytokines [31, 32]. Moreover, CCR7 and CD62L molecules play a key role during migration to secondary lymphoid organs [33]. When they act in lymphoid organs, Tregs are identified by the expression of inducible costimulatory (ICOS) or CD44 [34].

Different subtypes of Tregs show different states and varying functions. However, the conversion of Treg between different subtypes (activation) and the suppressive effect of Treg are dependent on cytokine transmission.

## 3. Activation and Suppressive Mechanism of Treg

### 3.1. Activation of Treg

**Figure 1:**
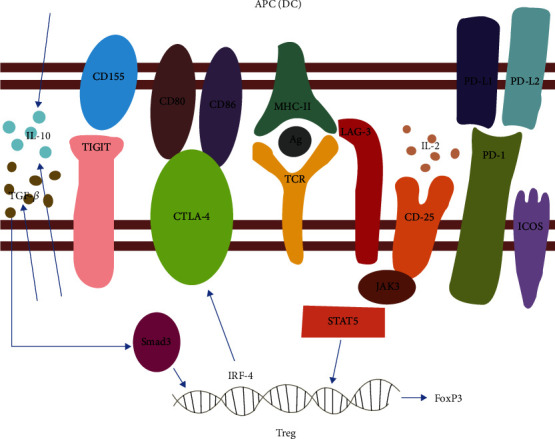
Cytokines and surface biomarkers of Treg and its interactions with APC. TCR binds to MHC-II/Ag and activates Treg differentiation, and LAG-3 negatively regulates it; CTLA-4, produced through activation of transcription factor IRF-4, binds to CD80/86 to suppress Th2 inflammation; PD-1 binds to PD-L1/PD-L2 to inhibit Teff cells and meanwhile enhance the transcriptional activation of Smad3 by TGF-*β*; TIGIT regulates the production of IL-10 and IL-12 by binding to CD155; CD25, receptor of IL-2, enables Treg to compete with Teff for IL-2 to proliferation.

On the surface, Tregs express the following: CD25, ITIM domain protein (TIGIT), lymphocyte activation gene 3 (LAG-3), ICOS [35–37], cytotoxic T lymphocyte antigen 4 (CTLA-4), tumor necrosis factor receptor (TNFR) family-related protein (GITR), programmed death-1 (PD-1), and its ligand (PD-L1) [38]. Tregs regulate the secretion of cytokines and the expression of surface biomarkers by expressing a variety of intracellular transcription factors, such as FOXP3 and IRF-4 [39]. They are responsible for Treg activation, function, and interaction with antigen-presenting cells (APCs) and other immune cells (summarized in Figure 1).

With antigens as an inflammatory stimuli, naïve CD4+ T cells can be induced and differentiated into Tregs [40]. Tregs usually act by regulating the recruitment of effector T cells to sites of inflammation and impairing the capacity of APCs that induce adaptive immune responses [41, 42]. Based on the expression of CD45RA or RO, Tregs can be divided into three subgroups: resting Tregs (rTregs, CD4^+^CD25^+^FOXP3^+^CD45RO^−^RA^+^), activated Tregs (aTregs, CD4^+^CD25^+^FOXP3^+^CD45RA^−^RO^+^), and cytokine-secreting nonsuppressive T cells (CD4^+^FOXP3^low^CD45RA^−^RO^+^ T cells) [43, 44]. Tumor necrosis factor receptor 2 (TNFR2) is highly expressed by aTregs and converts rTregs into an activated state [45, 46]. aTregs, which express elevated levels of TNFR2, appear to be highly immunosuppressive, and an excessive and uncontrolled expression of it may lead to cancer [47–49]. Meanwhile, cytokine-secreting nonsuppressive T cells produce proinflammatory cytokines, such as IL-17 [43].

The initiation and regulation of Tregs is modulated by APCs, such as dendritic cells (DCs) and macrophages. DCs are composed of conventional DCs (cDCs) and plasmacytoid DCs (pDC) [50]. The capacities of cDCs and pDCs to activate Tregs differ. pDCs usually have a poor capacity to induce Tregs but act as strong activators of effector T cells (Teff), which function differently from Tregs. Activated cDCs, which express higher levels of Toll-like receptor (TLR)-2,6,8 and self-peptide/MHC have the capacity to stimulate the proliferation and expansion of Tregs [51, 52]. CTLA-4 of Tregs can bind to ligands CD80 and CD86 on the surface of DCs to limit costimulatory signals [53]; PD-1 on Tregs can bind to PD-L1 and PD-L2, which are expressed on the surface of DCs to suppress the function of Teff [54].

Tregs can also proliferate by direct recognition of self-antigens and commensal microbes [55]. They can also clone and expand *in vivo* and *in vitro* after antigen stimulation and maintain their inhibitory function after expansion [56].

### 3.2. Cytokines Related to Treg

The activation of Tregs depends not only on surface biomarkers but also on cytokines. Further, Tregs are regulated by various networks of cytokines, such as IL-2, IL-7, IL-15, and TGF-*β*, to exert their immunosuppressive effects on T cells, B cells, NK cells, DCs, and macrophages.

IL-2 is important in regulating the development, function, and stability of Tregs [57]. IL-2 binds to CD25 of conventional T cells (Tconv) and activates three intracellular signaling pathways: mitogen-activated protein kinase (MAPK), phosphoinositide 3-kinase, and signal transducer and activator of transcription 5 [58]. These pathways regulate the proliferation and memory formation of Tconv as well as the immunosuppressive function of Tregs [58]. IL-2 directly upregulates FOXP3 and CD25, and when it binds to CD25, it lowers the threshold of effect on the development and homeostasis of Tregs [59–61]. Studies have also found that IL-7 can maintain the expression of CD25 and potentially enhance the responses of Tregs to IL-2, especially during cutaneous immunosuppression [32, 62, 63].

TGF-*β* is associated with the direction of differentiation of CD4+ T cells toward Tregs or Th 17 [57, 64]. TGF-*β* secreted by APCs or Treg itself can promote FOXP3 gene expression and lead to the differentiation of CD4+ T cells toward Tregs [57]. The downstream targets of TGF-*β* are mainly Smad family transcription factors, which interact with one another to form different types of DNA-binding complexes [65]. When exposed to lower concentrations of TGF-*β*, T cells can be induced to differentiate toward Th17 [40]. Despite the dual ability of TGF-*β*, some other cytokines (such as IL-2 and IL-6) are needed to further induce either FOXP3 or the transcription factor ROR_*γ*t_ [66–68]. For example, FOXP3 expression can be activated in the presence of TGF-*β* and IL-2 with TCR affinity and APC interaction [57].

IL-15 contributes to antigen presentation and the production of type I cytokines (IL-12 and IFN-*γ*) in APCs [69]. It is also involved in the stability and memory formation of CD4+ T cells [70]. In addition, IL-15 can promote FOXP3 expression via the STAT5 pathway [71]. Moreover, IL-15 acts as a supplement to IL-2 during the development of Tregs, but it cannot rescue the function of Treg in the absence of IL-2 [16]. Marshall et al. reported that IL-5 may provide an alternative pathway for CD4+ T cells to differentiate into Tregs, but this requires clarification [72].

In addition to the cytokines mentioned above, many other cytokines also regulate the function and behavior of Tregs. Tumor necrosis factor (TNF) can promote the proliferation of Tregs and regulate their suppressive function [73, 74]. IL-33, which promotes Th2-polarized immune responses, mediates the proliferation of Tregs and enhances their immune regulatory functions [75, 76]. IL-6 regulates the balance between Tregs and Th 17 by inhibiting the differentiation of Tregs and promoting Th 17 generation [77, 78].

## 4. Tregs in CRSwNP

**Figure 2:**
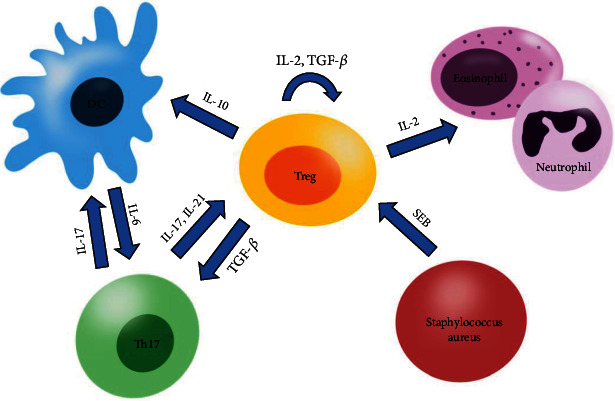
Interaction of different cytokines between cells in CRSwNP. In CRSwNP, Tregs secrete TGF-*β* to promote Th17 differentiation. Th17 inhibits Treg through IL-17 and IL-21, resulting in an imbalance between Treg and Th17 cells. APCs (such as DCs) activate Th17 cells via IL-6. SEB, secreted by Staphylococcus aureus, downregulates the number of Tregs. Meanwhile, Tregs produce IL-10 to inhibit the function of DCs and activate eosinophil/neutrophils by IL-2, and Treg cells also proliferate by autocrine IL-2 and TGF-*β*.

CRSwNP, the most common type of CRS, is thought to be associated with the Th2 immune response. It is usually characterized by the accumulation of eosinophils, T cells, neutrophils, and increased levels of IL-5 and IgE and decreased TGF-*β*1, which is in contrast to those of chronic rhinosinusitis without nasal polyps (CRSsNP) with elevated TGF-*β*1 and IFN-*γ* [79–81] (interactions between cells are shown in Figure 2; changes in cytokine levels from different studies are shown in Table 1). Studies have compared the differences between tissues from nasal polyps and the nasal sinus mucosa. The results showed that there was no significant difference in Tregs and CD4+ T cells between the two groups; however, elevated levels of pDCs and activated CD8+ T helper cells were observed in the nasal polyp tissue [82, 83]. Ickrath et al. [12] demonstrated that there was a significantly higher proportion of aTregs and memory T cells (CD3^+^ CD4^+^ CD45RA^−^ FOXP3^low^) in polyp tissues than in peripheral blood. Sharma et al. [30] conducted an immunohistochemical analysis of Tregs in the affected sinonasal tissues and found a large number of both CD25+ FOXP3+ and CD4+ FOXP3+ cells in the CRSwNP group than in the CRSsNP group (*p* < 0.05). Mann et al. reported that although there was a large proportion of Tregs in the polyp tissues, a lower proportion of aTregs was also observed in the CRSwNP group [84]. This explains why a larger number of Tregs result in a decreased immunosuppressive effect.

Impaired Treg function and differentiation play an important role in the onset and deterioration of CRSwNP. Treg exerts its suppressive effect mainly through its surface molecules, as well as the proteins (such as CD25 and CTLA-4) and cytokines it secretes (such as IL-10 and TGF-*β*). Treg dysfunction leads to the imbalance of Th1/Th2 and Treg/Th17 [85]. The hyperimmune response of Th2 results in IgE-mediated eosinophil infiltration and cellular edema, which promotes remodeling of the nasal sinus mucosa. Further, Treg/Th17 imbalance results in the imbalance between matrix metalloproteinases and tissue inhibitors of metalloproteinases via TGF-*β*1, and this causes the deposition of albumin, collagen, and other extracellular matrix proteins [86, 87]. Moreover, the decrease in Treg and downregulation of FOXP3 expression weaken the inhibition of Th17 differentiation, lead to the aggregation of neutrophils in the nasal sinus mucosa, and aggravate the inflammation characteristic of CRSwNP [88, 89].

However, there were geographical differences in the immune processes of CRSwNP. Studies from different regions have suggested that different races and geographical environments have an impact on the immune process of CRSwNP [93–96]. Some studies found that patients with CRSwNP from Western countries show more Th2-skewing inflammation, while patients from Southeast Asia tend to show Th1/Th17 inflammation [91, 97]. Cao et al. claimed that Chinese patients with CRSwNP showed reduced Tregs and decreased TGF-*β*1 production compared to healthy controls [79]. Similar conclusions were also observed by Li et al. [98] and Wang et al. [99].

The pathogenesis of CRSwNP is usually due to an imbalance of microbial flora (dysbacteriosis) in the nasal sinuses or the invasion of pathogenic microorganisms [100, 101], caused by many bacteria, such as *Corynebacterium tuberculstearicum*, *Staphylococcus aureus*, and *Haemophilus influenzae* [102–104]. Studies have reported that colonization by *Staphylococcus aureus* or *Haemophilus influenzae* potentially contributes to the development of nasal polyps [105, 106]. Studies have demonstrated that leukocidin ED, which is secreted by *Staphylococcus aureus* and acts as a cytolytic toxin, can result in the depletion of most effector memory T cells, leading to the impairment of Th1 and Th17 immune responses and disruption of balance between Th17 and Treg [107–109]. Staphylococcal enterotoxin B can trigger the significant elevation of IFN*γ*, IL-2, IL-13, etc. [110], which can also affect the differentiation and function of Tregs. In a study by Cho et al. [90], T cells that expressed retinoic acid receptor-related orphan receptor c (RORc) in peripheral blood mononuclear cells (PBMCs) significantly increased after treatment with Staphylococcal enterotoxin B in patients with eosinophilic polyps. Upregulation of RORc+ T cells can also downregulate Tregs [111]. Yang et al. also revealed the negative regulation of Tregs by *Haemophilus influenzae* after a long-term and low-dose exposure [112]. Rai et al. [113] investigated the changes in Th17, Treg, and various cytokines in patients with *Aspergillus flavus*-infected CRSwNP and found an immune imbalance with increased Th17, reduced Tregs, elevated levels of IL-10 and IL-17, decreased levels of TGF-*β* in PBMCs, and an elevated expression of TLR-2 in patients.

In patients with CRSwNP, the expression of FOXP3 is significantly lower than that in healthy controls [114]. FOXP3 acts as a biomarker of CD4+ Tregs and is important in regulating its function and development [115, 116]. In a study by Roongrotwattanasiri et al. [88], there was a decrease in the number of CD4+ Tregs; however, no difference was detected between the epithelia and lamina propria of nasal polyps and no difference between atopic and nonatopic CRSwNP patients. They inferred that the reduced expression of FOXP3 may lead to the enhancement of Th2 inflammation in both atopic and nonatopic patients and the high level of IgE based on the study by Pérez Novo et al. [117]. Ba et al. [118] also compared the inflammatory profiles between atopic and nonatopic patients with CRSwNP and found a decreased expression of FOXP3 and lower levels of Tregs irrespective of the atopic status. In addition, atopic CRSwNP patients produced more IL-5, IL-2, IL-10, IL-17A, and prostaglandin D2 in polyp tissues than nonatopic CRSwNP patients. Although the role of allergy in the process of CRSwNP is still in dispute, studies have found that CRSwNP caused by IgE sensitization to allergens can be an atopic disease [119–121].

The balance between Th17 and Treg cells plays a vital role in CRSwNP pathology [122]. These two subsets of T cells have opposite effects regarding autoimmune and inflammatory diseases [123–126]. However, the development of Th17 and Treg cells also shares reciprocal pathways: TGF-*β* can simultaneously activate the differentiation of both cells [77, 127, 128]; their transcription factors ROR*γ*t/ROR*α* and FOXP3 can inhibit each other's expression [129, 130]. A study by Li et al. [92] had pointed out that the expression level of ROR*γ*t was downregulated in patients with nasal polyposis, while the expression level of FOXP3 mRNA increased. Shen et al. also investigated the imbalance between Th17 and Treg and found a similar phenomenon to Li's study [122]. In patients with allergic fungal rhinosinusitis, Th17/Treg balance was also observed to be inclined toward Th17, which indicated atopy and aggravation of nasal polyposis [131].

In addition, the number of Tregs in PBMCs is negatively correlated with Th1- and Th2-related cytokines (such as INF-*γ*, IL-4, and IL-5) in polyp tissues. Although these changes appear in both atopic and nonatopic patients, they were more severe in atopic patients [122]. Chang et al. found that the adoptive transfer of Tregs can locally reduce the levels of proinflammatory cytokines and eosinophil cationic protein in nasal sinus mucosa to restore the balance between immune tolerance and effect in atopic patients [132]. Zheng et al. investigated the inflammatory profiles of pediatric antrochoanal polyps (ACP) and the effect of atopy on its pathogenesis and discovered that IL-10, a Treg-related cytokine, increased in patients with ACP and was positively correlated with IL-4 and IL-13. This indicated the regulatory role of Tregs in the inflammatory pathophysiological process of ACP [133]. Similar results were also observed in adult Chinese patients with ACP [134].

As for the reduced infiltration of Tregs in nasal polyp tissues, a study by Kim et al. demonstrated that there is no systemic defect in number but a defect in the migration capability of Tregs toward nasal epithelial cells [13]. Nasal epithelial cells can express CCL1 and CCL17, which can trigger chemotactic responses of Treg [135, 136]. However, in patients with CRSwNP, Tregs show low chemotactic responses to CCL1, decreased expression of CCL17, and altered levels of various cytokines that account for the reduction in the migration potential of Tregs toward nasal polyp tissues [137, 138].

## 5. Treg as Therapeutic Target in CRSwNP

Since the role of Treg in the pathogenesis of CRSwNP has been extensively studied, the study of Treg as a therapeutic target has also been widely investigated. Here, we list three nonsurgical treatments targeting Treg.

### 5.1. Steroid Therapy

In the treatment of CRSwNP, systemic or local administration of glucocorticoids (GCs), such as prednisone, which can suppress NF-*κ*B and MAPK pathways and alleviate tissue edema and nasal polyp size, was thought to be effective [139–141]. GCs have strong anti-inflammatory and immunomodulatory effects on immune cells and nonlymphoid tissues [142–145]. However, the effects of GCs on Tregs remain controversial. In terms of the impact on the number of Tregs, previous studies found that nonactivated Treg cells underwent apoptosis after administration of GCs [146]. Meanwhile, others reported of the relatively lower sensitivity of nonactivated Tregs to GC treatment that induced less apoptosis than other nonTreg cells [147, 148]. In addition, GC treatment can increase CTLA-4 cells and decrease CD69+ cells in the CD25+ cell population, which results in the expansion of Treg cells [149, 150]. Lin et al. investigated the effect of budesonide nasal spray treatment on CD8+ Tregs in patients with CRSwNP and found no increase in the percentage of CD8+ Treg cells in polyp tissues, although TGF-*β* and its mRNA were upregulated [151]. Edward et al. treated CRSwNP patients with oral prednisone and found an expansion of overlapped Treg cells in nasal polyp tissues via CCL4 chemotaxis without a significant increase in adjacent ethmoid sinus mucosa and PBMCs [152]. This conclusion confirms the results of earlier studies that Treg cells expanded locally in polyp tissues [153, 154]. However, in terms of the impact on the function of Tregs, some *in vitro* studies found that although the number of Treg cells significantly increased, their suppressive properties were not enhanced accordingly [155]. Nevertheless, it is still debatable whether the same results can be obtained in *in vivo* experiments. In the study by Kou et al., the nasal administration of GCs promoted the function of Tregs via the TGF-*β*1-Smad2 signaling pathway [154].

In terms of the impact on the differentiation of Tregs, the nasal administration of GCs was found to enhance TGF-*β*1 production to activate FOXP3 expression, which promoted the differentiation of T cells toward Tregs [154]. Moreover, TGF-*β*1 can drive IL-10 production, which can also maintain the expression of FOXP3, thus regulating the differentiation of Tregs [156].

### 5.2. Biological Therapy

Biologic therapy is becoming a novel and promising treatment for CRSwNP [157, 158]. Biologic drugs (monoclonal antibodies) can precisely block the effect of specific chemokines that control the Th2 inflammatory endotype when conventional therapy fails [159, 160]. Recently, many monoclonal antibodies targeting various cytokines have been developed, including omalizumab [161] (anti-IgE), mepolizumab [162, 163], reslizumab [164] (anti-IL-5), and dupilumab [165] (anti-IL-4 and IL-13). These targeted chemokines play key roles in the differentiation, chemotaxis, activation, and survival of eosinophils, basophils, mast cells, etc. [158]. Monoclonal antibodies targeting these chemokines have the ability to block different receptor-binding actions in patients with CRSwNP [157, 166, 167], which may alleviate the type 1 or type 2 inflammation response.

In addition to the monoclonal antibodies mentioned above that have been tested in patients with CRSwNP, other monoclonal antibodies targeting the Treg/Th17 balance have also been studied [168]. Some target Th17-related cytokines and receptors (such as IL-17, IL-23, and IL-6) that affect the expression level of ROR*γ*t indirectly increase Tregs in order to correct the imbalance [169–174]. Although no clinical trials have been conducted in CRSwNP patients, these still have the potential to improve CRSwNP symptoms by correcting the Treg/Th17 balance.

### 5.3. Probiotic Therapy

Based on the hypothesis of dysbacteriosis, probiotics are also used in the treatment of CRSwNP. Studies have investigated the therapeutic mechanisms of various bacteria, such as *Lactobacillus* and *Bifidobacterium*, as probiotics [175]. Previous studies have shown that *Lactobacillus* could regulate the differentiation of Th17 and Treg cells in peripheral lymphoid organs by mediating the production of several cytokines (such as IL-4 and IL-17) [176, 177]. Although the mechanisms of action of probiotics in CRSwNP treatment are not fully understood, Treg should be further studied as a medium for probiotics to modulate the immune response in CRSwNP.

## 6. Conclusion

Treg cells, a suppressive immunoregulatory T cell subset, play a vital role in the process of CRSwNP. CRSwNP, which is thought to be associated with the Th2 immune response, is characterized by Treg/Th17imbalance (decreased level of Tregs and increased level of Th17) in polyp tissue. Dysbacteriosis in the nasal sinuses or invasion of pathogenic microorganisms also contributes to the onset of CRSwNP. For example, *Staphylococcus aureus* can break the balance between Th17 and Treg cells, and Staphylococcal enterotoxin B can trigger the upregulation of RORc expression and downregulation of FOXP3 expression. However, further studies are needed to investigate the specific mechanism of dysbacteriosis interaction with Treg and other immune cells in order to better understand the immune modulation and disease progression of CRSwNP.

Th17 and Treg cells have opposite effects; however, their development also shares reciprocal pathways. The number of Tregs is negatively associated with Th1- and Th2-related cytokines (such as INF-*γ*, IL-4, and IL-5) in polyp tissues in both atopic and nonatopic patients. Future efforts should be made to understand the influence of atopy on the Treg/Th17 and Th1/Th2 balance in CRSwNP and their cytokine communication.

In the treatment of CRSwNP, glucocorticoids are considered a traditional and effective therapeutic drug because they potentially promote the expansion and function of Tregs. However, its effects on Tregs in different kinds of CRS need to be further studied. Monoclonal antibodies are a promising therapeutic option because they can block specific chemokines. However, at present, this therapy is relatively expensive, and monoclonal antibodies against Treg-related cytokines have not been confirmed to be effective in the clinical setting, which is still the direction of future efforts. Probiotics, such as Lactobacillus, are also used in the treatment of CRSwNP, but their effects on Tregs are not fully understood. Moreover, little is known about the effect of other probiotics in regulating Treg in CRSwNP. In the future, we should further investigate the role of probiotics in Treg immune regulation and identify more probiotics for Treg for the treatment of CRS. Additionally, through a deeper understanding of Treg and its related immune cells, safer and more effective novel therapeutic strategies need to be explored.

## Figures and Tables

**Table 1:** Changes of cytokines in patients with CRSwNP in different studies.

	Upregulated	Downregulated	Reference
Cho et al.	IL-2, IL-4, IL-6, IL-10, IL-17, and IFN-*γ*	nm	[90]
Ba et al.	IL-1*β*, IL-6, and IL-8	IL-5	[91]
Cao et al.	IFN-*γ*, IL-5, IL-17A, IL-22, and IL-23	nm	[79]
Li et al.	TGF-*β*, IL-10, and IL-18	IFN-*γ*	[92]
König et al.	IL-5, IL-17	IL-10, IL-12, IL-13, and IFN-*γ*	[81]

nm: not mentioned.

## Data Availability

All data generated or analyzed during this study are included in this article.

## References

[1] Rosenfeld R. M., Piccirillo J. F., Chandrasekhar S. S. (2015). Clinical practice guideline (update). *Otolaryngology--Head and Neck Surgery*.

[2] Fokkens W. J., Lund V. J., Hopkins C. (2020). EPOS2020: a major step forward. *Rhinology*.

[3] Philpott C., Erskine S., Hopkins C. (2016). A case-control study of medical, psychological and socio-economic factors influencing the severity of chronic rhinosinusitis. *Rhinology*.

[4] Kim J.-Y., Ko I., Kim M. S., Yu M. S., Cho B.-J., Kim D.-K. (2019). Association of chronic rhinosinusitis with depression and anxiety in a nationwide insurance population. *JAMA Otolaryngology–Head & Neck Surgery*.

[5] Soler Z. M., Eckert M. A., Storck K., Schlosser R. J. (2015). Cognitive function in chronic rhinosinusitis: a controlled clinical study. *International Forum of Allergy & Rhinology*.

[6] Van Crombruggen K., Zhang N., Gevaert P., Tomassen P., Bachert C. (2011). Pathogenesis of chronic rhinosinusitis: inflammation. *The Journal of Allergy and Clinical Immunology*.

[7] Pant H., Beroukas D., Kette F. E., Smith W. B., Wormald P. J., Macardle P. J. (2009). Nasal polyp cell populations and fungal-specific peripheral blood lymphocyte proliferation in allergic fungal sinusitis. *American Journal of Rhinology & Allergy*.

[8] Pant H., Macardle P. (2014). CD8(+) T cells implicated in the pathogenesis of allergic fungal rhinosinusitis. *Allergy & Rhinology*.

[9] Van Zele T., Gevaert P., Watelet J. B. (2004). Staphylococcus aureus colonization and IgE antibody formation to enterotoxins is increased in nasal polyposis. *The Journal of Allergy and Clinical Immunology*.

[10] Ickrath P., Scherzad A., Kleinsasser N., Ginzkey C., Hagen R., Hackenberg S. (2019). Influence of nasal polyp tissue on the differentiation and activation of T lymphocytes in a co-culture system. *Biomedical Reports*.

[11] Peters A. T., Kato A., Zhang N. (2010). Evidence for altered activity of the IL-6 pathway in chronic rhinosinusitis with nasal polyps. *Journal of Allergy and Clinical Immunology*.

[12] Ickrath P., Kleinsasser N., Ding X. (2017). Characterization of T-cell subpopulations in patients with chronic rhinosinusitis with nasal polyposis. *Allergy & Rhinology*.

[13] Kim Y. M., Munoz A., Hwang P. H., Nadeau K. C. (2010). Migration of regulatory T cells toward airway epithelial cells is impaired in chronic rhinosinusitis with nasal polyposis. *Clinical Immunology*.

[14] Lee G. R. (2018). The balance of Th17 versus Treg cells in autoimmunity. *International Journal of Molecular Sciences*.

[15] Dembic Z. (2008). Beginning of the end of (understanding) the immune response. *Scandinavian Journal of Immunology*.

[16] Sakaguchi S., Yamaguchi T., Nomura T., Ono M. (2008). Regulatory T cells and immune tolerance. *Cell*.

[17] Zhang X., Izikson L., Liu L., Weiner H. L. (2001). Activation of CD25(+)CD4(+) regulatory T cells by oral antigen administration. *Journal of Immunology*.

[18] Roychoudhuri R., Eil R. L., Restifo N. P. (2015). The interplay of effector and regulatory T cells in cancer. *Current Opinion in Immunology*.

[19] Noack M., Miossec P. (2014). Th17 and regulatory T cell balance in autoimmune and inflammatory diseases. *Autoimmunity Reviews*.

[20] Lou H., Fang J., Li P. (2015). Frequency, suppressive capacity, recruitment and induction mechanisms of regulatory T cells in sinonasal squamous cell carcinoma and nasal inverted papilloma. *PLoS One*.

[21] Bennett C. L., Christie J., Ramsdell F. (2001). The immune dysregulation, polyendocrinopathy, enteropathy, X-linked syndrome (IPEX) is caused by mutations of FOXP3. *Nature Genetics*.

[22] Hori S., Nomura T., Sakaguchi S. (2003). Control of regulatory T cell development by the transcription factor Foxp 3. *Science*.

[23] Komatsu N., Okamoto K., Sawa S. (2014). Pathogenic conversion of Foxp3^+^ T cells into T_H_17 cells in autoimmune arthritis. *Nature Medicine*.

[24] Shevyrev D., Tereshchenko V. (2020). Treg heterogeneity, function, and homeostasis. *Frontiers in Immunology*.

[25] Mills K. H. (2004). Regulatory T cells: friend or foe in immunity to infection?. *Nature Reviews Immunology*.

[26] Peng L. S., Zhuang Y., Shi Y. (2012). Increased tumor-infiltrating CD8(+)Foxp 3(+) T lymphocytes are associated with tumor progression in human gastric cancer. *Cancer Immunology, Immunotherapy: CII*.

[27] Robb R. J., Lineburg K. E., Kuns R. D. (2012). Identification and expansion of highly suppressive CD8+FoxP3+ regulatory T cells after experimental allogeneic bone marrow transplantation. *Blood*.

[28] Pant H., Hughes A., Schembri M., Miljkovic D., Krumbiegel D. (2014). CD4(+) and CD8(+) regulatory T cells in chronic rhinosinusitis mucosa. *American Journal of Rhinology & Allergy*.

[29] Abbas A. K., Benoist C., Bluestone J. A. (2013). Regulatory T cells: recommendations to simplify the nomenclature. *Nature Immunology*.

[30] Sharma S., Watanabe S., Sivam A. (2012). Peripheral blood and tissue T regulatory cells in chronic rhinosinusitis. *American Journal of Rhinology & Allergy*.

[31] Golubovskaya V., Wu L. (2016). Different subsets of T cells, memory, effector functions, and CAR-T immunotherapy. *Cancers*.

[32] Gratz I. K., Truong H. A., Yang S. H. (2013). Cutting edge: memory regulatory t cells require IL-7 and not IL-2 for their maintenance in peripheral tissues. *Journal of immunology*.

[33] Liston A., Gray D. H. (2014). Homeostatic control of regulatory T cell diversity. *Nature Reviews Immunology*.

[34] Dias S., D'Amico A., Cretney E. (2017). Effector regulatory T cell differentiation and immune homeostasis depend on the transcription factor Myb. *Immunity*.

[35] Georgiev P., Charbonnier L. M., Chatila T. A. (2019). Regulatory T cells: the many faces of Foxp 3. *Journal of Clinical Immunology*.

[36] Yu X., Harden K., Gonzalez L. C. (2009). The surface protein TIGIT suppresses T cell activation by promoting the generation of mature immunoregulatory dendritic cells. *Nature Immunology*.

[37] Gagliani N., Magnani C. F., Huber S. (2013). Coexpression of CD49b and LAG-3 identifies human and mouse T regulatory type 1 cells. *Nature Medicine*.

[38] Rosenblum M. D., Way S. S., Abbas A. K. (2016). Regulatory T cell memory. *Nature Reviews Immunology*.

[39] Zheng Y., Chaudhry A., Kas A. (2009). Regulatory T-cell suppressor program co-opts transcription factor IRF4 to control T_H_2 responses. *Nature*.

[40] Omenetti S., Pizarro T. T. (2015). The Treg/Th17 axis: a dynamic balance regulated by the gut microbiome. *Frontiers in Immunology*.

[41] Littman D. R., Rudensky A. Y. (2010). Th17 and regulatory T cells in mediating and restraining inflammation. *Cell*.

[42] Ryanna K., Stratigou V., Safinia N., Hawrylowicz C. (2009). Regulatory T cells in bronchial asthma. *Allergy*.

[43] Miyara M., Yoshioka Y., Kitoh A. (2009). Functional delineation and differentiation dynamics of human CD4^+^ T cells expressing the FoxP3 transcription factor. *Immunity*.

[44] Okubo Y., Torrey H., Butterworth J., Zheng H., Faustman D. L. (2016). Treg activation defect in type 1 diabetes: correction with TNFR2 agonism. *Clinical & Translational Immunology*.

[45] Chen X., Wu X., Zhou Q., Howard O. M., Netea M. G., Oppenheim J. J. (2013). TNFR2 is critical for the stabilization of the CD4+Foxp 3+ regulatory T. cell phenotype in the inflammatory environment. *Journal of Immunology*.

[46] Okubo Y., Mera T., Wang L., Faustman D. L. (2013). Homogeneous expansion of human T-regulatory cells via tumor necrosis factor receptor 2. *Scientific Reports*.

[47] Chopra M., Riedel S. S., Biehl M. (2013). Tumor necrosis factor receptor 2-dependent homeostasis of regulatory T cells as a player in TNF-induced experimental metastasis. *Carcinogenesis*.

[48] Govindaraj C., Scalzo-Inguanti K., Madondo M. (2013). Impaired Th1 immunity in ovarian cancer patients is mediated by TNFR2 \+ Tregs within the tumor microenvironment. *Clinical Immunology*.

[49] Govindaraj C., Tan P., Walker P., Wei A., Spencer A., Plebanski M. (2014). Reducing TNF receptor 2+ regulatory T cells via the combined action of azacitidine and the HDAC inhibitor, panobinostat for clinical benefit in acute myeloid leukemia patients. *Clinical Cancer Research*.

[50] Liu Y. J. (2005). IPC: professional type 1 interferon-producing cells and plasmacytoid dendritic cell precursors. *Annual Review of Immunology*.

[51] Hubo M., Jonuleit H. (2012). Plasmacytoid dendritic cells are inefficient in activation of human regulatory T cells. *PLoS One*.

[52] Yamazaki S., Iyoda T., Tarbell K. (2003). Direct expansion of functional CD25+ CD4+ regulatory T cells by antigen-processing dendritic cells. *The Journal of Experimental Medicine*.

[53] Qureshi O. S., Zheng Y., Nakamura K. (2011). Trans-endocytosis of CD80 and CD86: a molecular basis for the cell-extrinsic function of CTLA-4. *Science*.

[54] Chen L., Flies D. B. (2013). Molecular mechanisms of T cell co-stimulation and co-inhibition. *Nature Reviews Immunology*.

[55] Setoguchi R., Hori S., Takahashi T., Sakaguchi S. (2005). Homeostatic maintenance of natural Foxp 3(+) CD25(+) CD4(+) regulatory T cells by interleukin (IL)-2 and induction of autoimmune disease by IL-2 neutralization. *The Journal of Experimental Medicine*.

[56] Yamaguchi T., Hirota K., Nagahama K. (2007). Control of immune responses by antigen-specific regulatory T cells expressing the folate receptor. *Immunity*.

[57] Toomer K. H., Malek T. R. (2018). Cytokine signaling in the development and homeostasis of regulatory T cells. *Cold Spring Harbor Perspectives in Biology*.

[58] Cheng G., Yu A., Malek T. R. (2011). T-cell tolerance and the multi-functional role of IL-2R signaling in T-regulatory cells. *Immunological Reviews*.

[59] Burchill M. A., Yang J., Vogtenhuber C., Blazar B. R., Farrar M. A. (2007). IL-2 receptor beta-dependent STAT5 activation is required for the development of Foxp 3+ regulatory T cells. *Journal of Immunology*.

[60] Feng Y., Arvey A., Chinen T., van der Veeken J., Gasteiger G., Rudensky A. Y. (2014). Control of the inheritance of regulatory T cell identity by a cis element in the Foxp3 locus. *Cell*.

[61] Yu A., Zhu L., Altman N. H., Malek T. R. (2009). A low interleukin-2 receptor signaling threshold supports the development and homeostasis of T regulatory cells. *Immunity*.

[62] Harnaha J., Machen J., Wright M. (2006). Interleukin-7 is a survival factor for CD4+ CD25+ T-cells and is expressed by diabetes-suppressive dendritic cells. *Diabetes*.

[63] Simonetta F., Gestermann N., Bloquet S., Bourgeois C. (2014). Interleukin-7 optimizes FOXP3+CD4+ regulatory T cells reactivity to interleukin-2 by modulating CD25 expression. *PLoS One*.

[64] Schmidt A., Oberle N., Krammer P. H. (2012). Molecular mechanisms of treg-mediated T cell suppression. *Frontiers in Immunology*.

[65] Massagué J., Seoane J., Wotton D. (2005). Smad transcription factors. *Genes & Development*.

[66] Kleinewietfeld M., Hafler D. A. (2013). The plasticity of human Treg and Th17 cells and its role in autoimmunity. *Seminars in Immunology*.

[67] Konkel J. E., Chen W. (2011). Balancing acts: the role of TGF-*β* in the mucosal immune system. *Trends in Molecular Medicine*.

[68] Ueno A., Ghosh A., Hung D., Li J., Jijon H. (2015). Th17 plasticity and its changes associated with inflammatory bowel disease. *World Journal of Gastroenterology*.

[69] Ohteki T., Suzue K., Maki C., Ota T., Koyasu S. (2001). Critical role of IL-15-IL-15R for antigen-presenting cell functions in the innate immune response. *Nature Immunology*.

[70] Chen X. L., Bobbala D., Cepero Donates Y., Mayhue M., Ilangumaran S., Ramanathan S. (2014). IL-15 trans-presentation regulates homeostasis of CD4^+^ T lymphocytes. *Cellular & Molecular Immunology*.

[71] Vang K. B., Yang J., Mahmud S. A., Burchill M. A., Vegoe A. L., Farrar M. A. (2008). IL-2, -7, and -15, but not thymic stromal lymphopoeitin, redundantly govern CD4+Foxp 3+ regulatory T cell development. *Journal of Immunology*.

[72] Marshall D., Sinclair C., Tung S., Seddon B. (2014). Differential requirement for IL-2 and IL-15 during bifurcated development of thymic regulatory T cells. *Journal of Immunology*.

[73] Flores-Borja F., Jury E. C., Mauri C., Ehrenstein M. R. (2008). Defects in CTLA-4 are associated with abnormal regulatory T cell function in rheumatoid arthritis. *Proceedings of the National Academy of Sciences of the United States of America*.

[74] Mehta A. K., Gracias D. T., Croft M. (2018). TNF activity and T cells. *Cytokine*.

[75] Liew F. Y., Girard J. P., Turnquist H. R. (2016). Interleukin-33 in health and disease. *Nature Reviews Immunology*.

[76] Miller A. M. (2011). Role of IL-33 in inflammation and disease. *Journal of Inflammation*.

[77] Bettelli E., Carrier Y., Gao W. (2006). Reciprocal developmental pathways for the generation of pathogenic effector T_H_17 and regulatory T cells. *Nature*.

[78] Kimura A., Kishimoto T. (2010). IL-6: regulator of Treg/Th17 balance. *European Journal of Immunology*.

[79] Cao P. P., Li H. B., Wang B. F. (2009). Distinct immunopathologic characteristics of various types of chronic rhinosinusitis in adult Chinese. *Journal of Allergy and Clinical Immunology*.

[80] Polzehl D., Moeller P., Riechelmann H., Perner S. (2006). Distinct features of chronic rhinosinusitis with and without nasal polyps. *Allergy*.

[81] König K., Klemens C., Haack M. (2016). Cytokine patterns in nasal secretion of non-atopic patients distinguish between chronic rhinosinusitis with or without nasal polys. *Clinical Immunology*.

[82] Ho J., Bailey M., Zaunders J. (2015). Cellular comparison of sinus mucosa vs polyp tissue from a single sinus cavity in chronic rhinosinusitis. *International Forum of Allergy & Rhinology*.

[83] Cho K. S., Kim C. S., Lee H. S., Seo S. K., Park H. Y., Roh H. J. (2010). Role of interferon-*γ*-producing t cells in the pathogenesis of chronic rhinosinusitis with nasal polyps associated with staphylococcal superantigen. *Journal of Otolaryngology - Head & Neck Surgery*.

[84] Mann C., Schmidtmann I., Bopp T., Brieger J., Fruth K. (2020). Treg activation and their role in different subtypes of chronic rhinosinusitis. *Allergy*.

[85] Wu D., Song Q., Wang J. (2014). Study on the function and mechanism of Th17/Treg imbalance on mucosal remodeling of ECRSwNP. *Journal of Clinical Otorhinolaryngology, Head, and Neck Surgery*.

[86] Malinsky R. R., Valera F. C., Cavallari F. E. (2013). Matrix metalloproteinases and their impact on sinusal extension in chronic rhinosinusitis with nasal polyps. *European Archives of Oto-Rhino-Laryngology*.

[87] Van Bruaene N., Derycke L., Perez-Novo C. A. (2009). TGF-beta signaling and collagen deposition in chronic rhinosinusitis. *Journal of Allergy and Clinical Immunology*.

[88] Roongrotwattanasiri K., Pawankar R., Kimura S., Mori S., Nonaka M., Yagi T. (2012). Decreased expression of FOXP3 in nasal polyposis. *Allergy, Asthma & Immunology Research*.

[89] Palmer C., Mulligan J. K., Smith S. E., Atkinson C. (2017). The role of regulatory T cells in the regulation of upper airway inflammation. *American Journal of Rhinology & Allergy*.

[90] Cho S. N., Song C. H., Jin J., Kim S. H., Rha K. S., Kim Y. M. (2014). Role of staphylococcal enterotoxin B on the differentiation of regulatory T cells in nasal polyposis. *American Journal of Rhinology & Allergy*.

[91] Ba L., Zhang N., Meng J. (2011). The association between bacterial colonization and inflammatory pattern in Chinese chronic rhinosinusitis patients with nasal polyps. *Allergy*.

[92] Li C. W., Zhang K. K., Li T. Y. (2012). Expression profiles of regulatory and helper T-cell-associated genes in nasal polyposis. *Allergy*.

[93] Wang X., Zhang N., Bo M. (2016). Diversity of T_H_ cytokine profiles in patients with chronic rhinosinusitis: a multicenter study in Europe, Asia, and Oceania. *The Journal of Allergy and Clinical Immunology*.

[94] Lacroix J. S., Zheng C. G., Goytom S. H., Landis B., Szalay-Quinodoz I., Malis D. D. (2002). Histological comparison of nasal polyposis in black African, Chinese and Caucasian patients. *Chinese and Caucasian Patients Rhinology*.

[95] Luo B., Feng L., Jintao D. (2013). Immunopathology features of chronic rhinosinusitis in high-altitude dwelling Tibetans. *Allergy & Rhinology*.

[96] Seif F., Ghalehbaghi B., Aazami H. (2018). Frequency of CD4+ and CD8+ T cells in Iranian chronic rhinosinusitis patients. *Clinical Immunology*.

[97] Zhang N., Van Zele T., Perez-Novo C. (2008). Different types of T-effector cells orchestrate mucosal inflammation in chronic sinus disease. *The Journal of Allergy and Clinical Immunology*.

[98] Li X., Meng J., Qiao X. (2010). Expression of TGF, matrix metalloproteinases, and tissue inhibitors in Chinese chronic rhinosinusitis. *The Journal of Allergy and Clinical Immunology*.

[99] Wang Y., Wang Y., Ma Y., Pu X. (2016). The role of Th9, Th17 and Treg cells on pathogenesis of nasal polyps. *Journal of Clinical Otorhinolaryngology, Head, and Neck Surgery*.

[100] Copeland E., Leonard K., Carney R. (2018). Chronic rhinosinusitis: potential role of microbial dysbiosis and recommendations for sampling sites. *Frontiers in Cellular and Infection Microbiology*.

[101] Hoggard M., Wagner Mackenzie B., Jain R., Taylor M. W., Biswas K., Douglas R. G. (2017). Chronic rhinosinusitis and the evolving understanding of microbial ecology in chronic inflammatory mucosal disease. *Clinical Microbiology Reviews*.

[102] Abreu N. A., Nagalingam N. A., Song Y. (2012). Sinus microbiome diversity depletion and Corynebacterium tuberculostearicum enrichment mediates rhinosinusitis. *Science Translational Medicine*.

[103] Wagner Mackenzie B., Waite D. W., Hoggard M., Douglas R. G., Taylor M. W., Biswas K. (2017). Bacterial community collapse: a meta-analysis of the sinonasal microbiota in chronic rhinosinusitis. *Environmental Microbiology*.

[104] Ou J., Wang J., Xu Y. (2014). Staphylococcus aureus superantigens are associated with chronic rhinosinusitis with nasal polyps: a meta-analysis. *European Archives of Oto-Rhino-Laryngology*.

[105] Bose S., Grammer L. C., Peters A. T. (2016). Infectious chronic rhinosinusitis. *The Journal of Allergy and Clinical Immunology In practice*.

[106] Chalermwatanachai T., Vilchez-Vargas R., Holtappels G. (2018). Chronic rhinosinusitis with nasal polyps is characterized by dysbacteriosis of the nasal microbiota. *Scientific Reports*.

[107] Alonzo F., Kozhaya L., Rawlings S. A. (2013). CCR5 is a receptor for Staphylococcus aureus leukotoxin ED. *Nature*.

[108] Poddighe D., Vangelista L. (2020). Staphylococcus aureus infection and persistence in chronic rhinosinusitis: focus on leukocidin ED. *Toxins*.

[109] Spaan A. N., van Strijp J. A. G., Torres V. J. (2017). Leukocidins: staphylococcal bi-component pore-forming toxins find their receptors. *Nature Reviews Microbiology*.

[110] Martinez F. O., Helming L., Gordon S. (2009). Alternative activation of macrophages: an immunologic functional perspective. *Annual Review of Immunology*.

[111] Vickery T. W., Ramakrishnan V. R., Suh J. D. (2019). The role of Staphylococcus aureus in patients with chronic sinusitis and nasal polyposis. *Current Allergy and Asthma Reports*.

[112] Yang X., Wang Y., Zhao S., Wang R., Wang C. (2018). Long-term exposure to low-dose Haemophilus influenzae during allergic airway disease drives a steroid-resistant neutrophilic inflammation and promotes airway remodeling. *Oncotarget*.

[113] Rai G., Das S., Ansari M. A. (2020). TLR-2 expression and dysregulated human Treg/Th17 phenotype in Aspergillus flavus infected patients of chronic rhinosinusitis with nasal polyposis. *Microbial Cell Factories*.

[114] Van Bruaene N., Pérez-Novo C. A., Basinski T. M. (2008). T-cell regulation in chronic paranasal sinus disease. *Journal of Allergy and Clinical Immunology*.

[115] Curotto de Lafaille M. A., Kutchukhidze N., Shen S., Ding Y., Yee H., Lafaille J. J. (2008). Adaptive Foxp3^+^ regulatory T cell-dependent and -independent control of allergic inflammation. *Immunity*.

[116] Provoost S., Maes T., Van Durme Y. M. (2009). Decreased FOXP3 protein expression in patients with asthma. *Allergy*.

[117] Pérez Novo C. A., Jedrzejczak-Czechowicz M., Lewandowska-Polak A. (2010). T cell inflammatory response, Foxp 3 and TNFRS18-L regulation of peripheral blood mononuclear cells from patients with nasal polyps-asthma after staphylococcal superantigen stimulation. *Journal of the British Society for Allergy and Clinical Immunology*.

[118] Ba L., Du J., Liu F. (2015). Distinct inflammatory profiles in atopic and nonatopic patients with chronic rhinosinustis accompanied by nasal polyps in Western China. *Allergy, Asthma & Immunology Research*.

[119] Kennedy J. L., Borish L. (2013). Chronic sinusitis pathophysiology: the role of allergy. *American Journal of Rhinology & Allergy*.

[120] Krouse J. H. (2005). Allergy and chronic rhinosinusitis. *Otolaryngologic Clinics of North America*.

[121] Baba S., Kondo K., Toma-Hirano M. (2014). Local increase in IgE and class switch recombination to IgE in nasal polyps in chronic rhinosinusitis. *Journal of the British Society for Allergy and Clinical Immunology*.

[122] Shen Y., Tang X. Y., Yang Y. C. (2011). Impaired balance of Th17/Treg in patients with nasal polyposis. *Scandinavian Journal of Immunology*.

[123] Jiang Q., Yang G., Liu Q., Wang S., Cui D. (2021). Function and role of regulatory T cells in rheumatoid arthritis. *Frontiers in Immunology*.

[124] Maddur M. S., Miossec P., Kaveri S. V., Bayry J. (2012). Th17 cells: biology, pathogenesis of autoimmune and inflammatory diseases, and therapeutic strategies. *The American Journal of Pathology*.

[125] Selmi C. (2011). Autoimmunity in 2010. *Autoimmunity Reviews*.

[126] Li H., Wang Y., Wang J. (2021). Th17/Treg cells regulated by interleukin 6 in the pathogenesis of chronic rhinosinusitis with nasal polyps. *European Archives of Oto-Rhino-Laryngology*.

[127] Li M. O., Sanjabi S., Flavell R. A. (2006). Transforming Growth Factor-*β* Controls Development, Homeostasis, and Tolerance of T Cells by Regulatory T Cell-Dependent and -Independent Mechanisms. *Immunity*.

[128] Zhou L., Ivanov I. I., Spolski R. (2007). IL-6 programs T_H_-17 cell differentiation by promoting sequential engagement of the IL-21 and IL-23 pathways. *Nature Immunology*.

[129] Du J., Huang C., Zhou B., Ziegler S. F. (2008). Isoform-specific inhibition of ROR alpha-mediated transcriptional activation by human FOXP3. *Journal of Immunology*.

[130] Zhou L., Lopes J. E., Chong M. M. (2008). TGF-*β*-induced Foxp3 inhibits T_H_17 cell differentiation by antagonizing ROR*γ*t function. *Nature*.

[131] Rai G., Das S., Ansari M. A. (2018). Phenotypic and functional profile of Th17 and Treg cells in allergic fungal sinusitis. *International Immunopharmacology*.

[132] Chang L., Wang Z., Li S. (2020). Type 2 inflammation suppression by T-regulatory cells attenuates the eosinophil recruitment in mucosa of chronic sinusitis. *Clinical science*.

[133] Zheng H., Tang L., Song B. (2019). Inflammatory patterns of antrochoanal polyps in the pediatric age group. *Clinical Immunology*.

[134] Jin P., Zi X., Charn T. C. (2018). Histopathological features of antrochoanal polyps in Chinese patients. *Rhinology*.

[135] Terada N., Nomura T., Kim W. J. (2001). Expression of C-C chemokine TARC in human nasal mucosa and its regulation by cytokines. *Journal of the British Society for Allergy and Clinical Immunology*.

[136] Montes-Vizuet R., Vega-Miranda A., Valencia-Maqueda E., Negrete-García M. C., Velásquez J. R., Teran L. M. (2006). CC chemokine ligand 1 is released into the airways of atopic asthmatics. *The European Respiratory Journal*.

[137] Kim B., Lee H. J., Im N. R. (2018). Decreased expression of CCL17 in the disrupted nasal polyp epithelium and its regulation by IL-4 and IL-5. *PLoS One*.

[138] Park S. J., Kim T. H., Jun Y. J. (2013). Chronic rhinosinusitis with polyps and without polyps is associated with increased expression of suppressors of cytokine signaling 1 and 3. *The Journal of Allergy and Clinical Immunology*.

[139] Vaidyanathan S., Barnes M., Williamson P., Hopkinson P., Donnan P. T., Lipworth B. (2011). Treatment of chronic rhinosinusitis with nasal polyposis with oral steroids followed by topical steroids: a randomized trial. *Annals of Internal Medicine*.

[140] Badia L., Lund V. (2001). Topical corticosteroids in nasal polyposis. *Drugs*.

[141] Rhen T., Cidlowski J. A. (2005). Antiinflammatory action of glucocorticoids--new mechanisms for old drugs. *The New England Journal of Medicine*.

[142] Cain D. W., Cidlowski J. A. (2017). Immune regulation by glucocorticoids. *Nature Reviews Immunology*.

[143] Zielińska K. A., Van Moortel L., Opdenakker G., De Bosscher K., Van den Steen P. E. (2016). Endothelial response to glucocorticoids in inflammatory diseases. *Frontiers in Immunology*.

[144] Oppong E., Cato A. C. (2015). Effects of glucocorticoids in the immune system. *Advance Experimental Medicine Biology*.

[145] Gessi S., Merighi S., Borea P. A. (2010). Glucocorticoid's pharmacology: past, present and future. *Current Pharmaceutical Design*.

[146] Pandolfi J., Baz P., Fernández P. (2013). Regulatory and effector T-cells are differentially modulated by dexamethasone. *Clinical Immunology*.

[147] Li J., Wang Z., Hu S., Zhao X., Cao L. (2013). Correction of abnormal T cell subsets by high-dose dexamethasone in patients with chronic idiopathic thrombocytopenic purpura. *Immunology Letters*.

[148] Karagiannidis C., Akdis M., Holopainen P. (2004). Glucocorticoids upregulate FOXP3 expression and regulatory T cells in asthma. *The Journal of Allergy and Clinical Immunology*.

[149] Cari L., De Rosa F., Nocentini G., Riccardi C. (2019). Context-dependent effect of glucocorticoids on the proliferation, differentiation, and apoptosis of regulatory T cells: a review of the empirical evidence and clinical applications. *International Journal of Molecular Sciences*.

[150] Prado C., Gómez J., López P., de Paz B., Gutiérrez C., Suárez A. (2011). Dexamethasone upregulates FOXP3 expression without increasing regulatory activity. *Immunobiology*.

[151] Lin L., Dai F., Wei J., Chen Z. (2020). The role of budesonide on CD8^+^CD25^+^Foxp 3^+^ Treg cells in neutrophilic nasal polyps. *Journal of Clinical Otorhinolaryngology, Head, and Neck Surgery*.

[152] Edward J. A., Sanyal M., Le W. (2017). Selective expansion of human regulatory T cells in nasal polyps, and not adjacent tissue microenvironments, in individual patients exposed to steroids. *Clinical Immunology*.

[153] Li H. B., Cai K. M., Liu Z. (2008). Foxp3+ T regulatory cells (Tregs) are increased in nasal polyps (NP) after treatment with intranasal steroid. *Clinical Immunology*.

[154] Kou W., Hu G. H., Yao H. B. (2013). Transforming growth factor-*β*1 promotes Treg commitment in nasal polyposis after intranasal steroid treatment. *Inflammation Research*.

[155] Chung I. Y., Dong H. F., Zhang X. (2004). Effects of IL-7 and dexamethasone: induction of CD25, the high affinity IL-2 receptor, on human CD4^+^ cells. *Cellular immunology*.

[156] McGeachy M. J., Bak-Jensen K. S., Chen Y. (2007). TGF-*β* and IL-6 drive the production of IL-17 and IL-10 by T cells and restrain T_H_-17 cell-mediated pathology. *Nature Immunology*.

[157] Avdeeva K., Fokkens W. (2018). Precision medicine in chronic rhinosinusitis with nasal polyps. *Current Allergy and Asthma Reports*.

[158] Willson T. J., Naclerio R. M., Lee S. E. (2017). Monoclonal antibodies for the treatment of nasal polyps. *Immunology and Allergy Clinics of North America*.

[159] Prokopakis E., Vardouniotis A., Bachert C. (2020). Rhinology future debates 2018, a EUFOREA report. *Rhinology*.

[160] Hellings P. W., Akdis C. A., Bachert C. (2017). EUFOREA rhinology research forum 2016: report of the brainstorming sessions on needs and priorities in rhinitis and rhinosinusitis. *Rhinology*.

[161] Gevaert P., Calus L., Van Zele T. (2013). Omalizumab is effective in allergic and nonallergic patients with nasal polyps and asthma. *Journal of Allergy and Clinical Immunology*.

[162] Gevaert P., Van Bruaene N., Cattaert T. (2011). Mepolizumab, a humanized anti-IL-5 mAb, as a treatment option for severe nasal polyposis. *Journal of Allergy and Clinical Immunology*.

[163] Bachert C., Sousa A. R., Lund V. J. (2017). Reduced need for surgery in severe nasal polyposis with mepolizumab: randomized trial. *Journal of Allergy and Clinical Immunology*.

[164] Gevaert P., Lang-Loidolt D., Lackner A. (2006). Nasal IL-5 levels determine the response to anti-IL-5 treatment in patients with nasal polyps. *The Journal of Allergy and Clinical Immunology*.

[165] Bachert C., Mannent L., Naclerio R. M. (2016). Effect of subcutaneous dupilumab on nasal polyp burden in patients with chronic sinusitis and nasal polyposis: a randomized clinical trial. *Journal of the American Medical Association*.

[166] Caminati M., Senna G. (2019). Biologic therapy in a patient with asthma and nasal polyps. *The Journal of Allergy and Clinical Immunology In practice*.

[167] Hopkins C. (2019). Chronic rhinosinusitis with nasal polyps. *The New England Journal of Medicine*.

[168] Fasching P., Stradner M., Graninger W., Dejaco C., Fessler J. (2017). Therapeutic potential of targeting the Th17/Treg axis in autoimmune disorders. *Molecules*.

[169] Farahnik B., Beroukhim K., Abrouk M. (2016). Brodalumab for the treatment of psoriasis: a review of phase III trials. *Dermatology and Therapy*.

[170] Mease P. J., McInnes I. B., Kirkham B. (2015). Secukinumab inhibition of interleukin-17A in patients with psoriatic arthritis. *The New England Journal of Medicine*.

[171] Kavanaugh A., Puig L., Gottlieb A. B. (2016). Efficacy and safety of ustekinumab in psoriatic arthritis patients with peripheral arthritis and physician-reported spondylitis: post-hoc analyses from two phase III, multicentre, double-blind, placebo-controlled studies (PSUMMIT-1/PSUMMIT-2). *Annals of the Rheumatic Diseases*.

[172] Kikuchi J., Hashizume M., Kaneko Y., Yoshimoto K., Nishina N., Takeuchi T. (2015). Peripheral blood CD4(+)CD25(+)CD127 (low) regulatory T cells are significantly increased by tocilizumab treatment in patients with rheumatoid arthritis: increase in regulatory T cells correlates with clinical response. *Arthritis Research & Therapy*.

[173] Tada Y., Ono N., Suematsu R. (2016). The balance between Foxp 3 and Ror-*γ*t expression in peripheral blood is altered by tocilizumab and abatacept in patients with rheumatoid arthritis. *BMC Musculoskeletal Disorders*.

[174] Boyle D. L., Soma K., Hodge J. (2015). The JAK inhibitor tofacitinib suppresses synovial JAK1-STAT signalling in rheumatoid arthritis. *Annals of the Rheumatic Diseases*.

[175] Miyake M. M., Bleier B. S. (2019). Future topical medications in chronic rhinosinusitis. *International Forum Allergy Rhinology*.

[176] Chen R. C., Xu L. M., Du S. J. (2016). Lactobacillus rhamnosus GG supernatant promotes intestinal barrier function, balances T_reg_ and T_H_17 cells and ameliorates hepatic injury in a mouse model of chronic-binge alcohol feeding. *Toxicology Letters*.

[177] Shi C. W., Cheng M. Y., Yang X. (2020). Probiotic lactobacillus rhamnosus GG promotes mouse gut microbiota diversity and T cell differentiation. *Frontiers in Microbiology*.

